# EXPERIENCES WITH AND PREPAREDNESS FOR CLIMATE EMERGENCIES IN OREGON ASSISTED LIVING COMMUNITIES

**DOI:** 10.1093/geroni/igae098.0757

**Published:** 2024-12-31

**Authors:** Jacklyn Kohon, Dani Himes, Paula Carder, Ozcan Tunalilar

**Affiliations:** Portland State University, Portland, Oregon, United States; Portland State University, Portland, Oregon, United States; OHSU-PSU School of Public Health, Portland, Oregon, United States; Portland State University, Portland, Oregon, United States

## Abstract

Assisted living communities in the U.S. provide housing and care services to a sizable share of older adults and people with disabilities (over 900,000 individuals). The recent increase in climate-related emergencies has underscored the need for these communities to have preparedness measures in place to protect residents and staff. State agencies require preparedness plans, but these plans cannot anticipate all events. Oregon presents an important case study as a pioneer state in community-based care as well as with recent experience in various natural disasters. We interviewed administrators and management company operators (n=14), surveyed 140 direct care workers, and conducted focus groups with direct care workers (n=23) to learn about experiences during recent extreme heat events, wildfires, and ice storms. Thematic analysis revealed several themes related to the existing strengths and challenges in the current systems in place for responding to these emergencies. Strengths included an “all hands on deck” approach demonstrating teamwork among frontline workers, a commitment to show up for the residents in all conditions, and improved relationships with emergency management offices. Challenges included power and internet outage-related interruptions in technology for basic care delivery, insufficient plans for evacuation of residents with complex needs, increased staff injury, and lack of safe transportation. Our findings identify several opportunities and caution against pitfalls for regulators and policymakers in maintaining an acceptable level of emergency preparedness in AL settings.

